# Typhoid Fever1Read before the Medical Union, April 17, 1898.

**Published:** 1899-02

**Authors:** Walter D. Greene

**Affiliations:** Buffalo, N. Y., Deputy commissioner of health; clinical professor of genito-urinary diseases, Medical Department, University of Buffalo


### TYPHOID FEVER.[1]

By WALTER D. GREENE, M. D., Buffalo, N. Y.,
Deputy commissioner of health; clinical professor of genito-urinary diseases, Medical Department, University of Buffalo.

TYPHOID FEVER has been known and, in a way, has been studied for two centuries, but until the last twenty-five years, the cause had not been known. The last quarter of a century has been a period of great advancement along the lines of medical and surgical research, and the investigations into the causes of typhoid fever have been thorough and satisfactory. That it is largely disseminated through the agency of water is beyond question and is not contradicted.

When outbreaks of plague or cholera appear, with the loss of many lives, our attention is immediately fixed and our efforts directed to at once obliterate the disease as far as possible, but the mortality from typhoid fever is not generally noticed because it is always with us, not materially differing from year to year, and although causing many deaths, it is not viewed with as much alarm as would the same number of deaths occurring periodically.

Eberth discovered the bacillus of typhoid fever in 1880. Since that time it has been studied so thoroughly that its life history is well known. It finds abundant conditions in nature for its development, and is found in water, air, soiled clothing, dust, sewage and milk which has been contaminated with human intestinal discharges. It will grow readily at ordinary temperatures, but is killed by high temperatures. Another important fact is that the bacilli will retain their vitality for months when buried in the upper layers of soil. Cold does not materially affect them. Prof. Prudden kept them frozen in a block of ice for 103 days and yet when thawed he made colonies from them. They have been kept alive upon linen for two months and on buckskin and leather even longer. A two per cent. solution of carbolic acid will not kill them. They have been found in urine, blood and bowel discharges from typhoid fever patients. It is some-

1. Read before the Medical Union, April 17, 1898.

what uncertain how long after escape from the bowels the bacillus can be obtained, from the fact, that there are certain bacteria in normal fecal discharges which are inimical to the life of this special germ.

Therefore, an examination of sewage as it emerges from a hospital where there are numerous typhoid fever patients would reveal the presence of the bacilli at that point, yet a half mile away an examination might give negative results. The life range of the organism being so great and the use of water to the human economy so essential, it can be easily understood how readily water can be a carrier of the germ. In fact, the death-rate from typhoid fever for any given city can be taken as an index of the purity of its water supply. It must not be supposed that any given water or even sewage is teeming with pathogenic bacteria. They are found only in very small numbers as compared with other and harmless forms, and large quantities may be taken from pipes or reservoirs free from harmful bacteria, while just the cupful we take at a certain time may contain the pathogenic bacteria.

Another source of typhoid fever I believe to be connected with ground water and ground air, although this has been denied by many observers. There is a certain line or point in the ground where complete saturation is constant. Above this the earth may be dry, moist or saturated—these different conditions being controlled by the rainfall. In portions of the soil which are not well drained, the point of saturation would necessarily be near the surface. Now, suppose a case of typhoid fever occurred in an area similar to the one described and through ignorance or carelessness the discharge was thrown on the ground. If this occurred in the winter or spring, the special germ would lie practically dormant until the warm weather came on with a receding of the point of saturation in the ground—the place occupied by the water, being taken by air. Thus the conditions for the life and growth of the typhoid fever germ would be fulfilled—moisture, absence of sunlight, warmth. The special bacillus thrown upon the ground weeks before, thrives and multiplies under these favorable conditions. Now, when the autumn comes with its heavy rains, the point of saturation rises, the ground air loaded with bacteria is driven out and it is fair to suppose that some of the typhoid bacilli, assuming them to be present, would in a dessicated or semi-dessicated condition come in contact with food or water used by the people in the immediate vicinity. Should this theory prove to be correct, it would help to explain the cases occurring in low, badly-drained districts, where pure water is used.

Supposing that typhoid fever comes largely from the use of contaminated water, how can it be obviated?

The processes which tend to remove the contamination are oxidation, heat, sedimentation, nitrification and filtration. Of these, nitrification and oxidation are of little value. Sedimentation in quiet waters is of service as a large amount of solid matter sinks to the bottom in turbid waters, carrying with it numerous water organisms, but in rapidly moving streams little is accomplished. Heating water to boiling point will kill all bacteria contained in it, but it renders the water unpalatable by driving off the oxygen and carbonic acid gas and precipitating the salts of lime. Distillation will purify water as also will electrolysis. In summer, in exposed waters, the number of bacteria is much less than in winter. This is due to the sun's rays, which are very destructive to the water organisms and reduce their numbers very materially.

Buchner found that typhoid bacilli exposed on plates eight feet under water during bright sunshine were killed after four and one-half hours.

Filters for domestic use are generally useless or worse than useless, as many of them only arrest the larger particles, while the number of bacteria is added to by development in the filter. The Pasteur filter is one of the best, and all of the efficient filters are but modifications of it. It consists of a tube of unglazed porcelain, about one foot in length and an inch and a quarter in diameter, with walls one-eighth of an inch in thickness; one end is closed and the other opened for the passage of filtered water. This tube is placed upright in an enclosed metal case, into which the water from the main is allowed to enter. The normal pressure from the main will force the water through this one-eighth inch porcelain at the rate of about three gallons per hour. It is claimed, and the claim has been substantiated in several experiments, that this mode of filtration will remove about 96 to 98 per cent. of all bacteria. Other filters, although constructed on the same plan, filter through stone which, when clogged, can be removed, washed and put into an oven and heated to such a degree that all organisms are killed.

The porcelain tube in the Pasteur filter, is liable to be cracked by this procedure, and Dr. Thomas B. Carpenter, assistant bacteriologist of the health department, recommends that the tube be soaked in a 5 per cent. solution of calcic permanganate for half an hour, and then followed by soaking in a sodic sulphite solution.

That municipal filtration is the only safe remedy for polluted waters is fast becoming an accepted fact. There are two modes of filtration on a large scale. The one in use the longest is called the English filtering system, and it has been in use in England for many years, London having used it since 1839. It consists of a basin or several of them, covering about an acre each. Suitable drains for conveying away the filtered water are placed at the bottom, and these covered, first with coarse gravel and gradually becoming finer as the top is reached, which is covered with a coating of fine sand, about one and one-half feet in thickness; the whole bed of gravel and sand having a depth of about six feet. The water to be filtered is conducted into a large reservoir, where it is allowed to stand for from sixteen to twenty hours for sedimentation to take place, after which the upper half is allowed to run onto the filter bed. When first put into operation very little improvement is noticed in the quality of the filtered water. After several days the quality improves and not only does the water have an ocular appearance of purity, but examination reveals the fact that from ninety-six to ninety-nine per cent. of the bacteria has disappeared. Now, if this bed of the filtering basin be examined, it will be found that the upper layer of fine sand has arrested the larger particles found in the water and also that every grain of sand is covered with a living organic slime which is the principal cause of the efficiency of the filter.

This slime is composed of organisms of bacterial origin almost wholly. When, after some weeks, the filter becomes clogged with accumulations, and will not allow the water to run through fast enough to give the required amount of water, although what does pass through is nearly free from microörganisms, it is then necessary to draw off the water and scrape the superficial layer of fine sand to the depth of about three inches, when the filter is again ready for use. This upper layer of fine sand would consequently have to be renewed when the thickness was reduced to five or six inches. As I have said, this slime is composed of bacteria very largely. They are called the nitrifying organisms and many of them are identical with those which live in soil and decompose the organic matter in water. They are called nitrifying, because in breaking up the organic matter, they cause the nitrogen to unite with the oxygen in the water, forming harmless nitrates. If the above is true and this organic slime is the true arrester of the pathogenic and other bacteria, it would follow that if the bed was disturbed so that the slime was removed there would be danger of infection.

This has been found to be the cause in Hamburg and Berlin where the bed had been disturbed by the removal of ice in winter, and mild epidemics of typhoid fever ensued—large quantities of bacteria being found in the water coming from the filters. In climates where the temperature is low during certain months of the year it has been found necessary to cover the basins, which precludes the danger of freezing and also in great part prevents the growth, during the hot months, of algæ in the basins, which give to certain waters a very unpleasant taste.

The cost of covered filters in Europe has been estimated at about $75,000 per acre, which ought to give about 2,500,000 gallons of pure water every twenty-four hours.

The second method is called the American or mechanical plan of filtration. By this mode, filtration is about ten times as fast as in the English and is dependent upon the formation of an inorganic slime upon the small grains of sand instead of an organic. This is formed by adding to the water, as it goes into the filter, from two-thirds to one grain of alum per gallon. This is decomposed by the lime in the water forming an aluminum hydrate, which settles on the sand at the bottom of the filtering basin. Many experiments have been made tending to show that under proper conditions the results are as good as in the English system. The advantage claimed for this system are that it is cheaper in construction and does not require as much space. The filters are cleaned by reversing the flow of water until the flow is clear. The estimated cost of this plan is about 10 per cent. less than the English, but it costs more to operate it. I thus give a very cursory description of the method of filtration adapted to large cities, and now a word as to their efficiency.

Hudson, N. Y., takes her water from the Hudson, into which, above the intake, the sewage of 409,000 people is emptied. She has used sand filtration for twenty years and her annual average death-rate from typhoid fever for nine succeeding years was 22 per 100,000 population.

Lawrence, Mass., is situated on the Merrimac river, from which it takes its water supply. There are fifteen cities and towns situated on the river above the intake with a total population something over 250,000. The sewage from these towns empties into the river. The average annual death-rate from typhoid fever in Lawrence, per 100,000 population, for the six years preceding the establishing of a sand filter, in 1893, was 111. January 1, 1894, the water was being regularly filtered and the death-rate that year from typhoid fever, per

100,000, 40.7, for 1895, 30.1, and 1896, 10.9, showing a steady decrease from year to year.

I have not the death-rate for 1897 at hand. Certain of these deaths occurring after the filtering plant was in operation were caused by some of the employees in the mills drinking unfiltered water from the river. These are two instances out of many that show how human life can be saved by proper means.

Now as to Buffalo. The average annual death-rate from typhoid fever in our city, per 100,000 population, was 60 in 1894; 32 in 1895; 19½ in 1896; 22 in 1897, and for the three months this year, January, February and March, something over 31. That is not a bad showing as compared with other cities using raw water, and yet with the increase of population and the increased liability of water contamination, the rate is liable to grow unless the water is properly filtered.

The health department is taking all the precautions known to sanitary science to keep down the death-rate, but there are factors to contend with that cannot be controlled by municipal authority.

In September, 1895, there occurred 115 cases of typhoid fever in Buffalo as against 51 and 24 in the corresponding months of 1896 and 1897, an increase of over 100 per cent. in one case and nearly 500 in the other. The undoubted cause was an outbreak of typhoid fever along the banks of Grand river in Canada, which empties into Lake Erie, about forty miles above Buffalo. This was at its height in August and the conditions were favorable for the typhoid bacillus to float down the lake and river and to be taken into our water pipes. Not since 1894 have there been so many cases of typhoid reported in March as occurred last month—fifty cases, with eleven deaths. March, 1895, there were reported four cases of typhoid; in March, 1896, 20; March, 1897, 9, and March, 1898, 50.

This, I believe, to have been due to the thaws and freshets in February, when Buffalo river overflowed its banks, bringing all sorts of débris down from the country, spreading over large areas of manured land, and running far out into the harbor—far enough for some of it to be taken up at the inlet pier and pumped into our houses.

Filtering plants cost money and need strict attention, and to be efficient the water should be bacteriologically examined daily, but the value of human life cannot be computed by dollars and cents. A proper filtering plant established in Buffalo would result in the saving of from 50 to 100 lives annually from typhoid fever alone.

385 Jersey Street.

